# A Randomized Controlled Feasibility Trial in Behavioral Weight Management for Underserved Postpartum African American Women: The RENEW Study

**DOI:** 10.5888/pcd15.170400

**Published:** 2018-06-14

**Authors:** Priya P. Joshi, Lisa M. Quintiliani, Ashley C. McCarthy, Ashley Gilmore, Mufaddal Mahesri, Lisa M. Sullivan, Caroline M. Apovian

**Affiliations:** 1 Boston Medical Center, Section of General Internal Medicine, Boston, Massachusetts; 2 Boston University School of Medicine, Medical Information Systems Unit, Boston, Massachusetts; 3 Boston Medical Center, Section of Endocrinology, Diabetes and Nutrition and Weight Management, Boston, Massachusetts; 4 Indiana University Department of Medicine, Division of Gastroenterology and Hepatology, Indianapolis, Indiana; 5 Boston University School of Public Health, Department of Biostatistics, Boston, Massachusetts

## Abstract

We aimed to test the feasibility of an in-person behavioral weight-loss intervention for underserved postpartum African American women with overweight or obesity in an urban hospital setting. Participants were randomized to an intervention of a culturally tailored adaptation of the Diabetes Prevention Program or usual care. The primary outcome was program satisfaction. Women who completed the intervention reported higher levels of satisfaction with the program, despite low attendance rates at group meetings. The intervention was not feasible because of these low rates of attendance and high rates of attrition after randomization. Offering the program electronically and off-site for convenience and more psychosocial support for postpartum women with obesity may improve feasibility.

## Objective

Most (82.1%) non-Hispanic African American women aged 20 years or older in the United States are overweight (body mass index [BMI] ≥25.0 kg/m^2^), and 56.7% are obese (BMI ≥30.0 kg/m^2^) (1), predisposing them to abnormal gestational weight gain during pregnancy, postpartum weight retention (2), and increased risk for cardiovascular disease (3). African American women are disproportionately affected by postpartum weight retention compared with white women (4). To address this racial health disparity, we studied the feasibility of an adaptation of the Intensive Lifestyle Intervention from the Diabetes Prevention Program (5) for at-risk women from low-income communities. We hypothesized that a culturally tailored behavioral weight-loss program would be feasible and would reduce postpartum weight retention in African American women.

## Methods

This study, Revolutionizing Exercise and Nutrition Everyday in Women (RENEW), was a randomized controlled trial conducted at Boston Medical Center from August 2011 through September 2013. After an initial visit, postpartum women were randomly assigned to an intervention group or a usual-care group. The intervention was an 8-session culturally tailored, in-person, group-based weekly weight-loss program, focusing on supervised exercise (resistance training or dancing) and nutrition education (Box); intervention participants were also partnered with a peer health worker (Birth Sister) for moral support and assistance. The usual-care group received one counseling visit with a study dietitian and an educational brochure on weight loss. Both groups were asked to attend a final assessment visit 8 weeks after the initial visit; we defined completers as women who attended the initial visit and the final visit. Our primary outcome of interest was program satisfaction. We administered surveys at the final visit to collect data on program satisfaction; attendance was recorded at each session, and weight was measured at the initial and final visits. We also collected data via questionnaire on changes in eating behavior, a secondary outcome of interest. The study was approved by the Boston University Medical Center Institutional Review Board.

**Box. Lesson Plans From a Randomized Controlled Feasibility Trial in Behavioral Weight Management for Underserved Postpartum African American Women: the Revolutionizing Exercise and Nutrition Everyday in Women (RENEW) Intervention Study, 2011–2013**

**Table:** 

Lesson Theme and Content	Take-Home Items
Lesson 1 — Introduction: review of physical activity and caloric intake guidelines, and review of lactation to augment weight loss	Portion-size placemat, a soul-food pyramid handout, resistance bands with instruction for use at home, and scheduling for future sessions
Lesson 2 — Review of exercising using targeted heart rate for weight loss	25-minute supervised salsa dancing and 10-minute resistance-band lesson
Lesson 3 — Detailed review of healthy carbohydrates, seizing opportunities for increasing daily physical activity, identifying environmental cues that trigger hunger and how to change them	DVD, “Dance Off the Inches: Sizzling Salsa”; measuring cups; and affordable food resources handout
Lesson 4 — Review of low-saturated-fat diet, difference between good and bad fats, and fat-calorie monitoring	Mrs. Dash samples
Lesson 5 — Cooking healthy meals at home, using the Boston Medical Center demonstration kitchen dietitian; attention was drawn to a season-specific topic (ie, fiber, whole grains, good vs bad fats, increasing fruit and vegetable intake, etc) by using culturally relevant and readily available foods	$25 grocery store gift card and recipes from the meals prepared in the demonstration
Lesson 6 — Coping with postpartum stress and emotional eating, taught by a licensed independent clinical social worker from Boston Medical Center with review of a handout on emotional-eating diagram; the 35-minute salsa dancing and resistance band training were repeated	None
Lesson 7 — How to “eat out”: planning ahead, assertion, stimulus control, navigating a restaurant menu for healthy options, and portion control; the 35-minute salsa dancing and resistance band training were repeated	None
Lesson 8 — Lifestyle-change pitfalls and how to anticipate and avoid them; individual weight-loss progress review with dietitian; the 35-minute salsa dancing and resistance band training were repeated	New food log (for use after intervention), sample food labels, participant-specific calorie goal (based on Mifflin St-Jeor formula), pediatric nutrition handout

Underserved African American women aged 18 or older receiving prenatal care at Boston Medical Center were screened for overweight and obesity, estimated delivery date, and readiness to change. We categorized patients as underserved according to characteristics such as race, ethnicity, geography, and health outcomes (6). Inclusion criteria included self-identified African American race, preconception BMI of 25.0 or more, contemplative stage of change (7), and English-speaking. Exclusion criteria were signs of moderate-to-severe depression based on the Patient Health Questionnaire-9 (8), enrollment in the Birth Sisters program before the study, and nonviable birth (Figure). Prestudy sample size was 30 in each arm. Randomization to each group was calculated by using a 1:1 allocation based on permuted blocks of size 4. We used 2 independent sampled *t* tests to compare mean age and BMI measured at baseline between the randomized groups and χ^2^ or Fisher exact tests to compare program satisfaction and eating patterns between the randomized groups. To assess intervention effects, we used independent 2-sample *t* tests to compare weight gain from baseline to the end of the study between the randomized groups.

**Figure:**
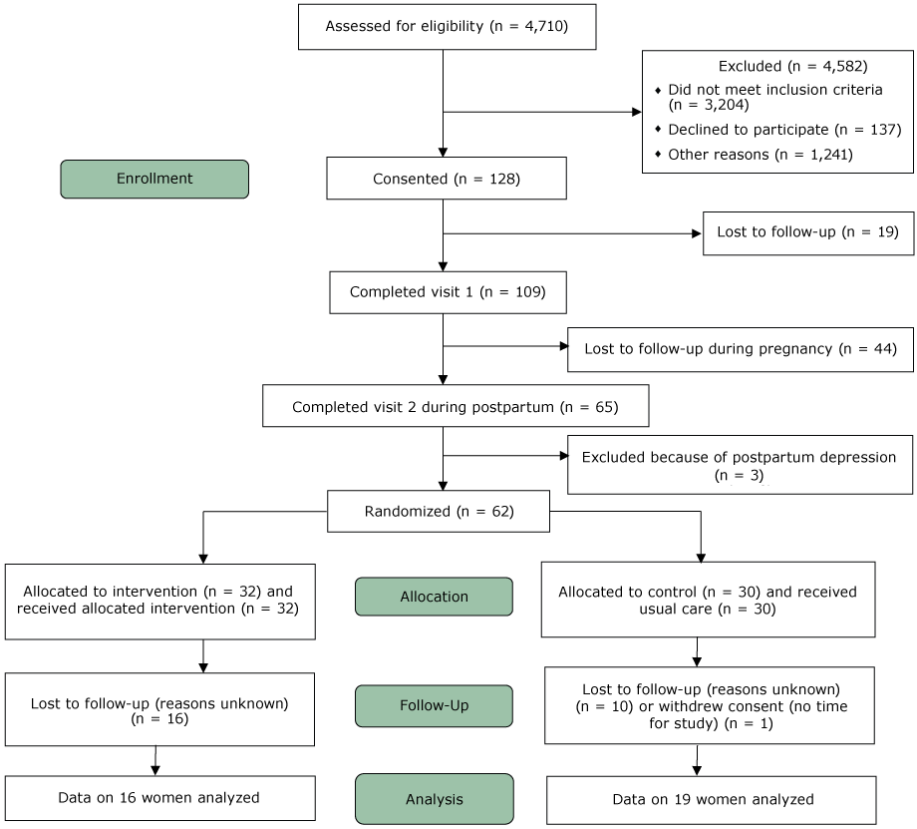
CONSORT diagram of the progress through the phases of a randomized controlled feasibility trial in behavioral weight management for underserved postpartum African American women, Boston, Massachusetts, 2011–2013.

## Results

Of the 128 women who provided informed consent, 62 were randomized to the intervention or usual-care group. Overall, participant characteristics for both groups were similar at baseline. The mean (standard deviation [SD]) age was 30.2 (6.1) years in the intervention group and 31.2 (6.3) years in the usual-care group (*t* = 0.63, *P *= .53). Median (interquartile range) income was $12,500 ($2,500–22,500) in the intervention group and $17,500 ($2,500–30,000) in the usual-care group. Mean (standard deviation [SD]) BMI was 35.6 (7.7) in the intervention group and 34.4 (5.3) in the usual-care group (*t* = 0.72, *P* = .49). Sixteen of 32 (50%) women in the intervention group and 19 of 30 (63%) women in the usual-care group completed the final assessment visit.

Mean attendance in the intervention group was 40%: 11 women attended no group sessions, 12 women attended 1 to 5 sessions, and 9 women attended 6 sessions or more. Compared with participants in the usual-care group, intervention participants reported greater enjoyment of the weight-loss program (58% vs 100%, respectively, enjoyed “very much”) (χ^2 ^= 8.3, *P *= .004), thought the program met their diet and exercise needs and weight-loss goals (26% vs 60%, respectively, thought so “very much”) (χ^2^ = 3.9, *P* = .048), and thought the program should be continued at the hospital (58% vs 93%, respectively, thought “definitely yes”) (χ^2^ = 5.4, *P* = .47). Both groups showed an increase in low-fat eating (intervention, mean [SD] = 4.7 [2.4] vs usual care, mean [SD] 4.2 [1.8], *P* = .86). Both groups gained weight (intervention, mean = 2.4 kg, *t* = 1.83, *P* = .09; usual care, mean = 2.2 kg, *t* = 2.48, *P* =.02) by the end of the study, but the difference in weight change between the intervention and usual-care groups was not significant (*t* = .14, *P* = .89). Weight change among intervention group participants who attended 6 or more sessions (n = 12) was not significantly different from weight change in the usual-care group (*t* = .31, *P *= .76).

## Discussion

Nearly half of pregnant women in the United States exceed recommended weight gain during pregnancy, most of whom have preconception obesity (9). Cultural perceptions of health are important considerations for successful weight-loss interventions. African American women have reported larger body size and uninhibited pregnancy-related weight gain as healthful for themselves and their babies (10,11). Several studies of African American women have articulated fear of judgment, inadequate psychosocial support, and positive association with curvaceous body appearance as impediments to weight loss (4,12).

The primary feasibility outcome of our study was program satisfaction, which was rated highly by intervention participants. In addition, the increases in low-fat eating among women in the intervention group were greater than the increases among women in the usual-care group.

Both groups gained weight, regardless of the number of sessions attended, suggesting natural progression of postpartum weight retention. However, our adaptation of the Intensive Lifestyle Intervention may not have been tailored adequately for our study population, and this lack of cultural specificity may explain the study’s low rate of attendance. Formative research that explores our study population’s preferences for activity and anticipated barriers to program attendance could yield a more tailored weight-loss intervention. Anecdotal reports from study participants highlighted child care and transportation as key obstacles to program attendance. An internet-based intervention showed promise with postpartum weight loss (13). Because our program was offered only in person at Boston Medical Center, accessibility was a limitation of our study. Thus, integrated approaches, shifting program delivery to community settings, using community health worker outreach in conjunction with digital health tools as part of an electronically disseminated and supervised program, are indicated.

For sustained postpartum weight loss in underserved African American women who reduce weight after birth, future interventions should begin during preconception, be culturally specific, accessible, and garner community support.
